# SR-FTIR as a tool for quantitative mapping of the content and distribution of extracellular matrix in decellularized book-shape bioscaffolds

**DOI:** 10.1186/s12891-018-2149-9

**Published:** 2018-07-18

**Authors:** Yongchun Zhou, Can Chen, Zhu Guo, Shanshan Xie, Jianzhong Hu, Hongbin Lu

**Affiliations:** 1 Department of Sports Medicine, Xiangya Hospital, Central South University, 87# Xiang-ya Road, Changsha, 410008 Hunan People’s Republic of China; 2 Department of Spine Surgery, Xiangya Hospital, Central South University, 87# Xiang-ya Road, Changsha, 410008 Hunan People’s Republic of China; 3 Key Laboratory of Organ Injury, Aging and Regenerative Medicine of Hunan Province, Changsha, China; 4 Research Centre of Sports Medicine, Xiangya Hospital, Central South University, Changsha, China; 5 Xiangya Hospital-International Chinese Musculeskeletal Research Society Sports Medicine Research Centre, Changsha, China

**Keywords:** SR-FTIR, Decellularized bioscaffolds, Regeneration, Bone-tendon interface

## Abstract

**Background:**

To evaluate synchrotron radiation-based Fourier transform infrared microspectroscopy (SR-FTIR) as a tool for quantitative mapping of the content and distribution of the extracellular matrix in decellularized fibrocartilage bioscaffolds, and to provide a new platform for quantitatively characterizing bioscaffolds for tissue engineering.

**Methods:**

Fibrocartilage was harvested and cut into book-shape bioscaffolds (*N* = 54), which were then decellularized. The structures and distribution of collagen fibrous and intrinsic ultrastructure in decellularized fibrocartilage bioscaffolds were evaluated by histological staining and scanning electron microscopy (SEM), respectively. The content of collagen and proteoglycan in the cellularized or decellularized bioscaffolds were also measured by SR-FTIR and biochemical assay.

**Results:**

Book-shape fibrocartilage decellularized bioscaffolds were successfully obtained. Histological examination revealed that the structure of extracellular matrix endured during decellularization. Histology and DNA quantification analysis confirmed substantial removal of cells during decellularization. SEM demonstrated that intrinsic ultrastructure of the fibrocartilage bioscaffold was also well preserved. SR-FTIR quantitative analysis confirmed that decellularization had a significant effect on the content and distribution of collagen and proteoglycan in fibrocartilage bioscaffolds, these results are confirmed with the biochemical assay results.

**Conclusion:**

SR-FTIR imaging can capture the histological morphology of decellularized bioscaffolds. Moreover, it can be used for quantitative mapping of the content and distribution of collagen in the bioscaffolds.

## Background

Bone-tendon interface injuries caused by sports, surgery and trauma are extremely common, and represent a huge burden on medical resources [1]. The bone-tendon interface has a unique transitional structure with region-specific distributions in cell type and matrix composition [2]. This unique structure can anchor the bone and tendon together depending on the fibrous fibrocartilage zone, thus dispersing stress at the bone-tendon interface and balancing conduction of the mechanical load between the bone and tendon [3]. This unique tissue structure often fails to regenerate during healing [4, 5]. Traditional treatments for bone-tendon interface injuries involve direct attachment to the bone using surgical techniques. However the bone-tendon interface is often filled with fibrovascular scars, and reconstruction of the original characteristic fibrocartilage zone fails to regenerate the gradient structure. Therefore, the tendon-bone interface may fail to transfer load efficiently between the soft and hard tissues, which can lead to unable to heal eventually [6]. To improve bone-tendon interface healing, in recent years researchers have applied cytokines, biophysical stimulation, stem cell therapy and other methods to aid repair of bone-tendon interface injuries. Nevertheless, the damaged bone-tendon interface cannot completely recover the original graded transitional structure [7].

Decellularized bioscaffolds have received great attention in the tissue engineering field allowing reconstruction of damaged tissues or organs due to their ability to enhance tissue regeneration and repair damaged tissues [8–10]. As the repair of bone-tendon interface injuries requires replication of the original graded transitional structure, decellularized bioscaffolds may prove useful in this application. At present, the traditional method of two-dimensional histological section staining is still used to evaluate the extracellular matrix of decellularized bioscaffolds. These traditional techniques can evaluate the distribution of extracellular matrix, but fail to quantitatively analyze the extracellular matrix components of the biological tissue.

Several studies have been showed that Fourier transform infrared microspectroscopy (FTIR) can assess chemical composition of extracellular matrix [11–13]. This technology can overcome the inherent shortcomings of histology, e.g., batch-to-batch variations in staining solutions and qualitative interpretation of stains. In recent years, with the development of science and technology, synchrotron radiation-based Fourier transform infrared microspectroscopy (SR-FTIR) has become a hot topic for the analysis of complex components in biological samples [14]. With higher resolution and improved signal-to-noise ratio (SNR) compared to conventional FTIR, the quantity, composition, structure and distribution of the chemical components in the sample can be analyzed, in combination with the extraction of more detailed structural information [15–17], displaying significant advantages of the chemical composition of even small samples. The extracellular matrix of decellularized bioscaffolds is composed of different functional components, and whether or not these components are affected in the process of decellularization has not been determined. So far, the application of SR-FTIR for quantitative evaluation of extracellular matrix components in decellularized bioscaffolds has not been reported.

This study aimed to apply SR-FTIR as a tool for quantitative mapping of the content and distribution of extracellular matrix in the cellularized or decellularized bioscaffolds, so as to provide a new platform for quantitative evaluation of extracellular matrix in decellularized bioscaffolds.

## Methods

### Preparation of fibrocartilage samples

**Fig. 1:**
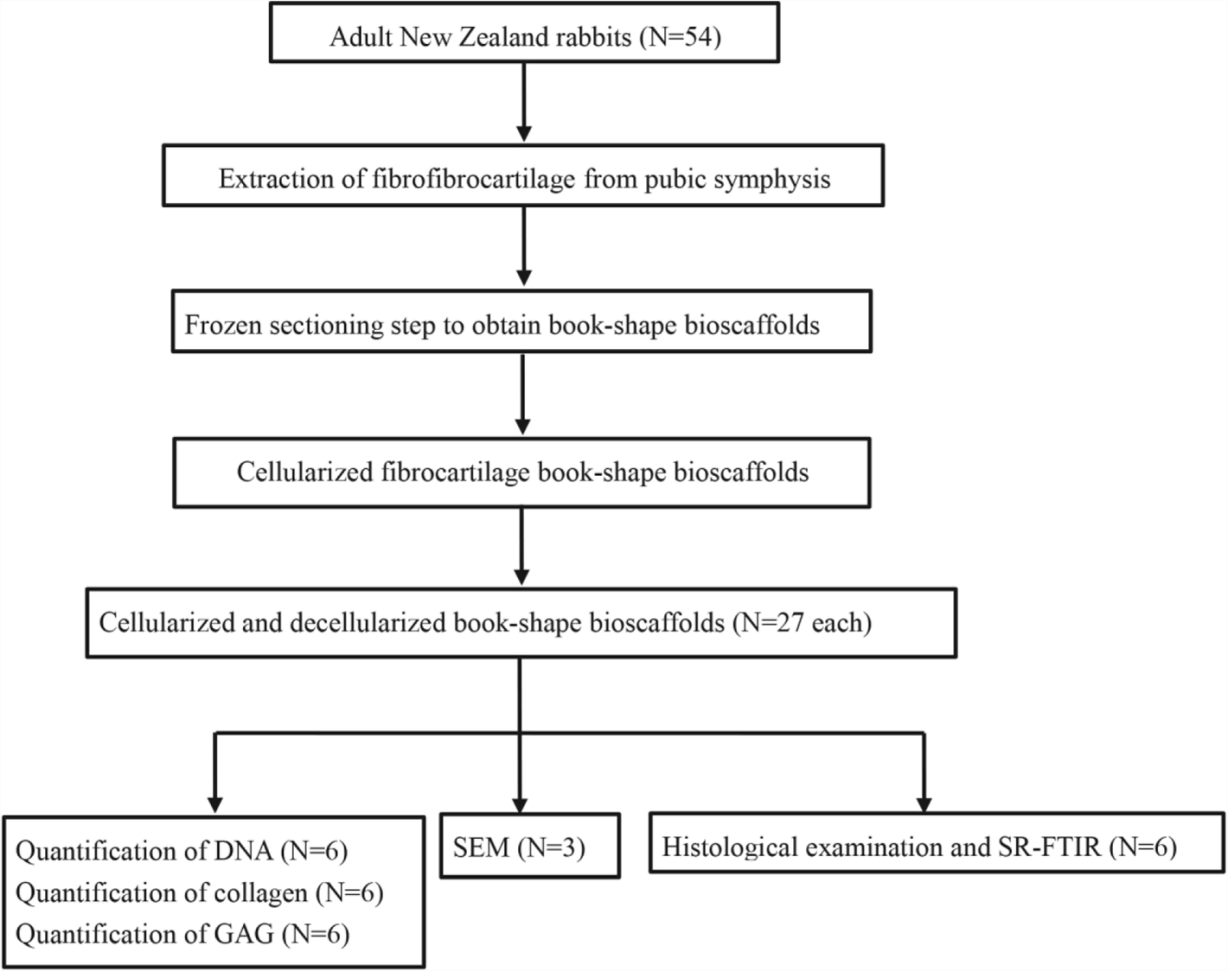
The flow chart of the preparation of decellularized book-shape fibrocartilage bioscaffolds

The experiment design is illustrated as in Fig. 1. A total of 54 female New Zealand rabbits (18 weeks old, 2.9 ± 0.2 kg) (supplied by the Experimental Animal Center of Central South

University, Changsha, China) were euthanized (intraperitoneal injection of sodium pentobarbital, 100 mg/kg) and the fibrocartilage samples were harvested from the pubic symphysis. Following the removal of bone and soft tissues around the fibrocartilage, fibrocartilage was cut into blocks of about 7 mm × 2 mm × 1 mm in size. All experimental procedures conformed to the guidelines in the Guide for the Care and Use of Laboratory Animals published by the Chinese National Health and were approved by the Ethics Committee of the Center for Scientific Research with Animal Models of Central South University (2013–3-13).

### Preparation of cellularized and decellularized “book” fibrocartilage bioscaffolds

To prepare cellularized specimens, the trimmed fibrocartilage samples were embedded in the optimum cutting temperature (OCT) compound (polyvinyl alcohol polyethylene glycol, Tissue-Tek1; Sakura Finetek USA, Inc., Torrance, CA, USA), and were placed for 20 min at − 22 °C first before freeze-sectioning using cryostat (Leica CM1950; Nussloch, Germany). Subsequently, the samples were sliced to a thickness of 100 μm to obtain book-shape fibrocartilage specimens with only one end cut apart. Then, the book-shape fibrocartilage scaffolds were washed in deionized water three times for 30 min each time, to remove OCT embedding agent.

The decellularized book-shape fibrocartilage bioscaffolds were prepared as previously described with modifications [10, 18]. Following the removing of OCT, the book-shape fibrocartilage scaffolds were incubated with 2% SDS for 2 h at 37 °C with vigorous agitation, the fibrocartilaginous bioscaffolds were washed with PBS and incubated with a nuclease solution containing 500 U/mL DNase Type I and 50 mg/mL RNase with agitation at 37 °C for 12 h. Samples were then treated with 0.02% EDTA for 24 h after nuclease digestion, followed by PBS washing (× 6 cycles, in 8 h). Furthermore, by applying a vacuum freeze-drier (Virtis Benchtop 6.6, SP Industries, Gardiner, NY), lyophilization was performed in the experimental samples. In the above steps, 100 mg/mL streptomycin, 100 U/mL penicillin, 2.5 mg/mL fungizone and aprotinin (10 KU/mL) were added to all solutions although aprotinin was omitted from the nuclease solution. Finally, the book-shape bioscaffolds were washed with deionized water and embedded in paraffin, then cut into 5 μm slices for histological, and SR-FTIR examinations.

### Histology and scanning electron microscopy (SEM) analysis

Samples for histological detection and SR-FTIR were fixed immediately for 24 h with 80% ethanol supplemented with 1% cetylpyridinium chloride (CPC, Sigma, St. Louis, MO, USA) to minimize the effect of fixation on the IR spectral parameters and to preserved proteoglycans, then washed in deionized water [11]. Hematoxylin and eosin (H&E) stain was used to detect the structures and distribution of collagen fibrous, and 4,6-diamidino-2-phenylindole (DAPI) (Sigma-Aldrich, USA) was used to detect the cellular components.

The samples used for SEM scanning were fixed with 2.5% glutaraldehyde for 24 h and washed with PBS, and then washed with PBS, dehydrated by gradient ethanol, and soaked with isoamyl acetate. The microstructure of the bioscaffold surface was observed under SEM (Hitachi S-3400 N, Japan) after drying and spraying.

### Synchrotron radiation-based fourier transform infrared spectroscopy (SR-FTIR) analysis

The effect of decellularization on the extracellular matrix components of bioscaffolds was evaluated using SR-FTIR. Sample preparation and FTIR spectral analysis were performed as described previously [19, 20]. Specifically, paraffin embedded bioscaffolds were sagittally sectioned into 5 μm slices, which were placed rapidly onto BaF2 substrate (Spectral Systems, Hopewell Junction, USA). Sections were then dried overnight under vacuum following dewaxing and dehydration. Furthermore, another BaF2 substrate was applied in the bioscaffolds before infrared analysis. Synchrotron radiation was collected from a bending magnet, collimated and transported to a commercial FTIR interferometer bench. After modulation by the interferometer, a commercial infrared microscope focused the beam on to the sample using all-reflecting optics. The sample was placed on the sample stage and the sample stage position was controlled by a computer. The reflected light from the sample was collected by the microscope optics and sent to an IR detector. A computer performed a Fourier transform on the measured interferogram to obtain an infrared spectrum for each sample location. To characterize the content and distribution of collagen and proteoglycan in the bioscaffolds, SR-FTIR spectromicroscopy was performed with the BL01B beam line at SSRF (Shanghai Synchrotron Radiation Facility). Nicolet Continuum XL microscope (Thermo Fisher Scientific) equipped with a 250 × 250 μm^2^ liquid nitrogen cooled MCT/A detector, a 32X/NA0.65 Schwarzschild objective, a motorized knife-edge aperture, and a Prior XYZ motorized stage and coupled with Nicolet 6700 spectrometer (ThermoFisher) equipped with a Michelson interferometer was included to record spectra with a spectral and spatial resolution of 8 cm^− 1^ resolution and 5 μm, respectively. The background was collected through a blank substrate. Prior to analysis, spectra were background corrected by baseline subtraction. Chemical maps were created and analyzed with the Omnic software (Thermo Fisher). The distribution of collagen and proteoglycan was mapped using the amide I (1720 to 1590 cm^− 1^) and carbohydrate (1140–985 cm^− 1^), respectively. Collagen content was estimated by integrating the peak area under the Amide I band, and proteoglycan content was estimated by integrating the area under a carbohydrate band [12, 13]. Three areas of each sample were selected randomly for imaging and analysis.

### Quantifications of DNA, collagen, and GAG in the fibrocartilage bioscaffolds by biochemical assay

The quantity of DNA, collagen, and GAG in cellularized and decellularized bioscaffolds were calculated as previously described [10]. Following decellularization, the fibrocartilage bioscaffolds samples (*n* = 6 for each group) were frozen at − 80 °C for 12 h. Next, the samples were freeze-dried at − 70 °C for 24 h using a lyophilizer (SIM International Group, USA), then weighed and minced. The quantifications of DNA, collagen, and GAG were performed using the DNeasy Blood & Tissue protocol (Qiagen, Germany), Blyscan collagen assay kit (Biocolor, UK), and A Sircol GAG assay (Biocolor), respectively, according to the manufacturer’s instructions.

### Statistical analysis

SPSS13.0 statistical software (SPSS Inc) was used for statistical analysis, and all data were expressed as mean ± SD values. Statistical significance of the experimental variables was then evaluated using Student’s t-test (*P* < 0.05).

## Results

### Characterization of decellularized book-shape fibrocartilage bioscaffolds by histological and SEM analysis

**Fig. 2:**
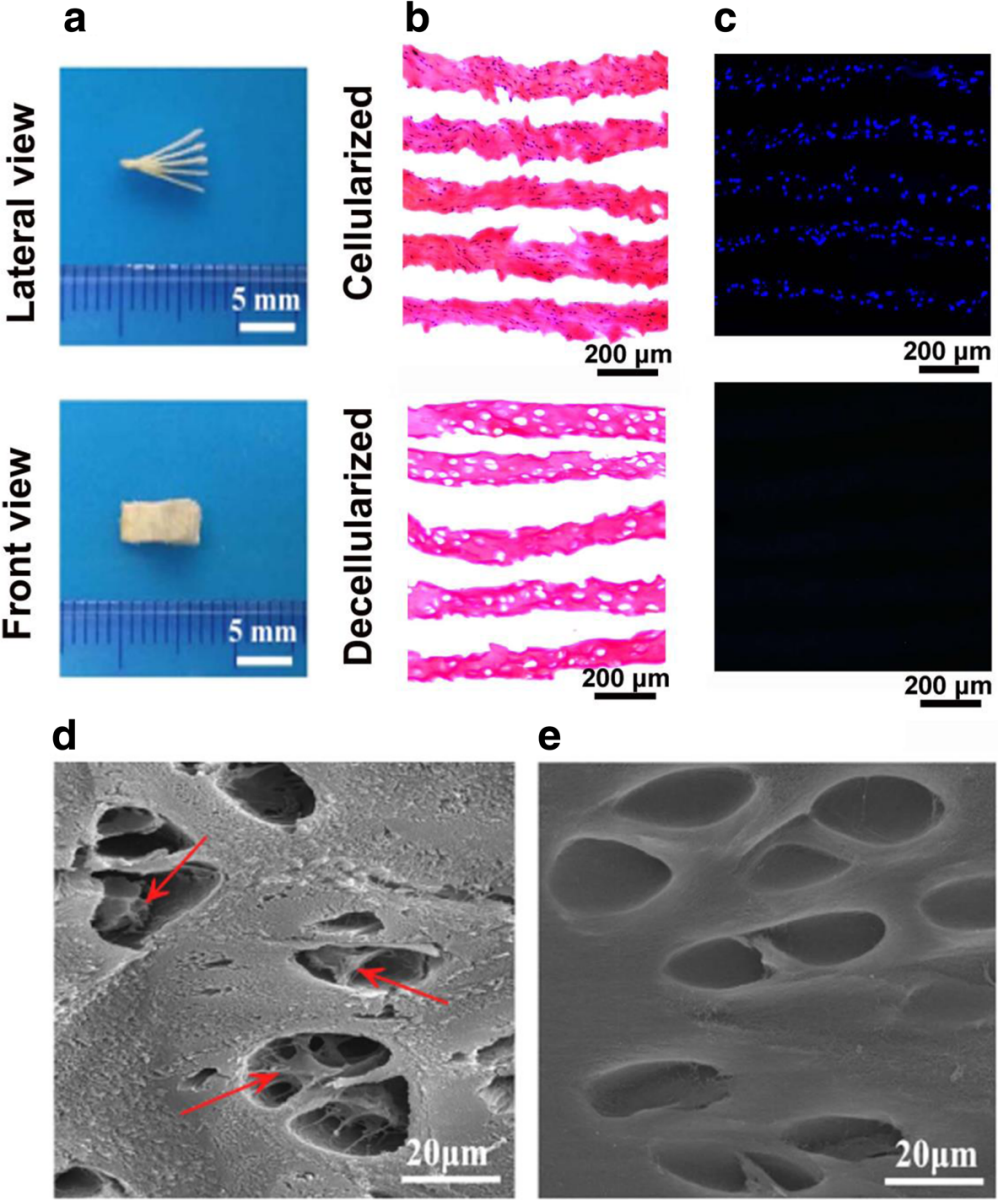
Gross, histological and SEM observation of cellularized and decellularized bioscaffolds. Gross observation and size measurement of book-shape fibrocartilage decellularized bioscaffolds (**a**); HE staining (**b**) and DAPI staining (**c**) revealed that cell components were removed and the structure of extracellular matrix bioscaffolds was retained in the decellularized fibrocartilage bioscaffolds; Cells (indicated by arrow) were found adhered to fibrocartilage lacuna under SEM in the cellularized bioscaffolds (**d**); No fibrocartilage cells were found in the decellularized bioscaffolds, with visible characteristic fibrocartilage lacuna and native collagen structure (**e**)

The book-shape fibrocartilage bioscaffolds (7 mm × 2 mm × 0.5 mm) were prepared as shown in Fig. 2a. HE staining suggested that the structure of extracellular matrix bioscaffolds was retained following decellularization, but cellular components were removed (Fig. 2b). DAPI-positive cell nuclei were rarely observed following decellularization (Fig. 2c). The microstructure of cellularized or decellularized bioscaffolds was shown by SEM (Fig. 2 d and e). Cells were adhered to the fibrocartilage lacuna in the cellularized bioscaffolds (Fig. 2d). After decellularization, no cell adhesion was observed in the bioscaffolds, but the characteristic fibrocartilage lacuna and native collagen structure were well preserved (Fig. 2e).

### Contents and distributions of collagen and carbohydrate by SR-FTIR

**Fig. 3:**
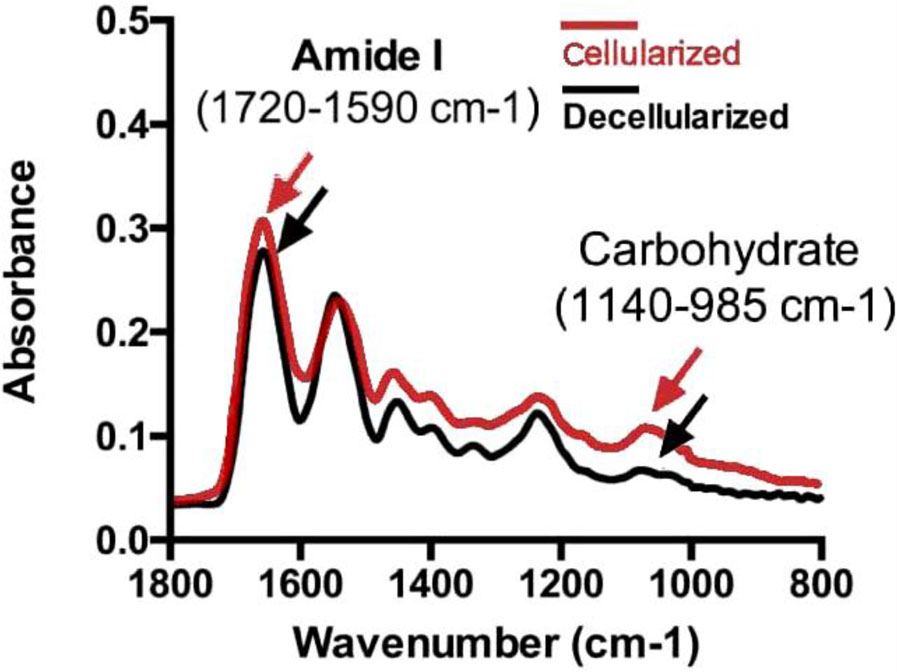
Representative infrared spectra: Red color line indicates cellularized bioscaffolds; Black color line indicates decellularized bioscaffolds. Note: Peaks of amide I and carbohydrate were indicative for collagen and proteoglycan, respectively

Representative infrared spectra of cellularized or decellularized bioscaffolds by SR-FTIR were shown in Fig. 3. Peaks of amide I (1720–1590 cm^− 1^) and carbohydrate (1140–985 cm^− 1^) were present in both cellularized (Red color line) and decellularized bioscaffolds (Black color line).

**Fig. 4:**
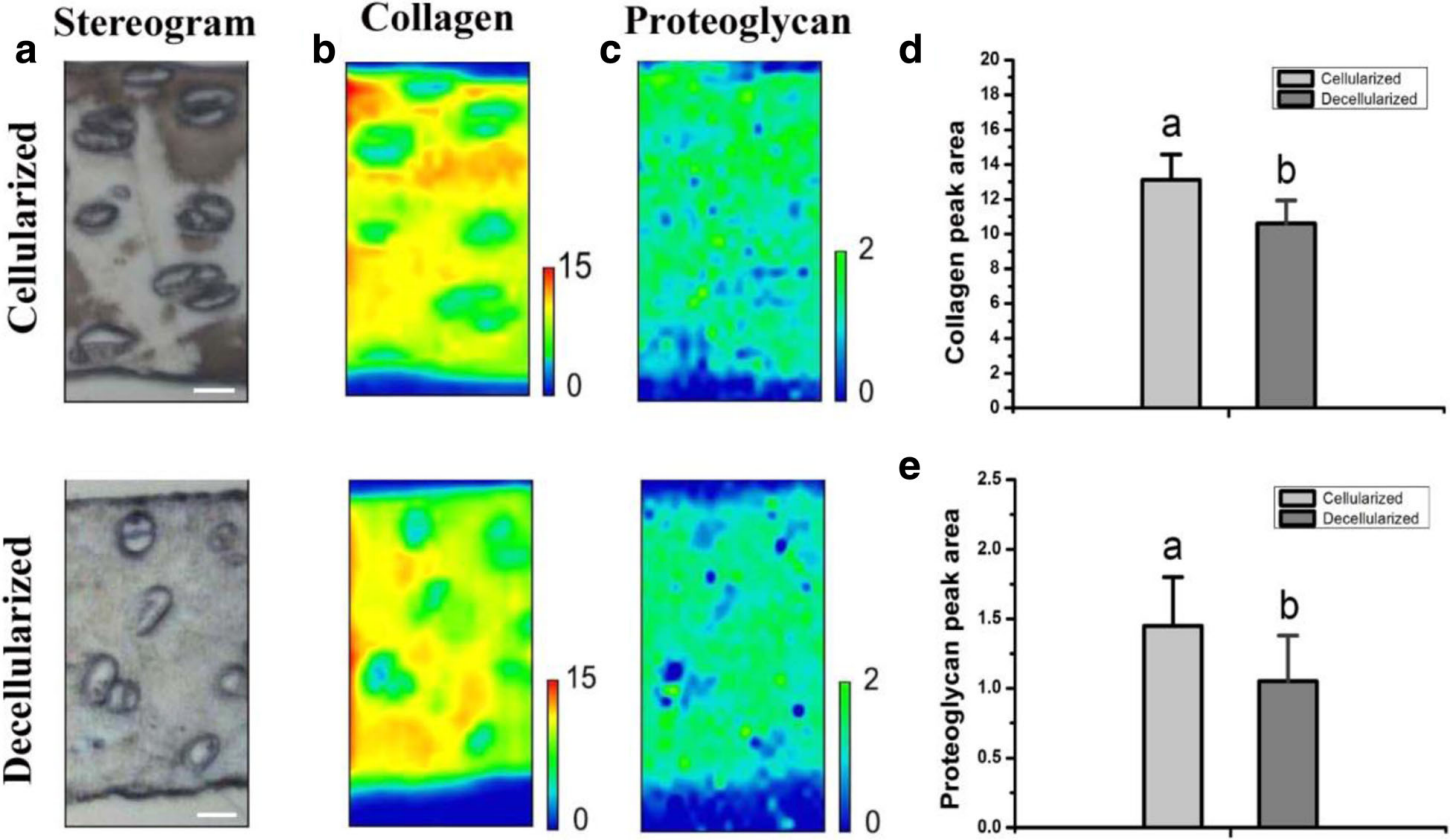
Analysis of the content and distribution of collagen in transverse sections of bioscaffolds. **a** Light microscopy images of cellularized or decellularized bioscaffolds in the collected IR spectra (bar = 10 μm). **b** Spectroscopic maps of the distribution and content of collagen in the cellularized and decellularized bioscaffolds. Red and blue colors indicate high and low matrix content, respectively. **c** Spectroscopic maps of the distribution and content of proteoglycan in the cellularized and decellularized bioscaffolds. Green and blue colors indicate high and low matrix content, respectively. **d** Content of collagen within the cellularized or decellularized bioscaffolds. **e** Content of proteoglycan within the cellularized or decellularized bioscaffolds. Dissimilar letters indicating a significant difference (*P* < 0.05)

Both cellularized and decellularized bioscaffolds in the collected IR spectra were shown in the stereogram by light microscopy (Fig. 4a). The intensity distribution of collagen (amide I) and proteoglycan (carbohydrate) were measured. Maps were plotted using the absorption spectra (minimum and maximum absorption were expressed in blue and red or blue and green, respectively). Spectroscopic maps of the collagen distribution (Fig. 4b) and proteoglycan distribution (Fig. 4c) were obtained for cellularized or decellularized bioscaffolds. Compared to the cellularized scaffold, the collagen and proteoglycan distribution in the decellularized scaffold was similar, but the content of the collagen (13.12 ± 1.45 vs 10.61 ± 1.32, *p* < 0.05) and proteoglycan (1.45 ± 0.35vs 1.05 ± 0.33, *p* < 0.05) was reduced significantly after the cells were removed (Fig. 4d and e). 19.1% collagen and 27.6% proteoglycan is lost during decellularization.

### Content of DNA, collagen, and GAG by biochemical assay

**Fig. 5:**
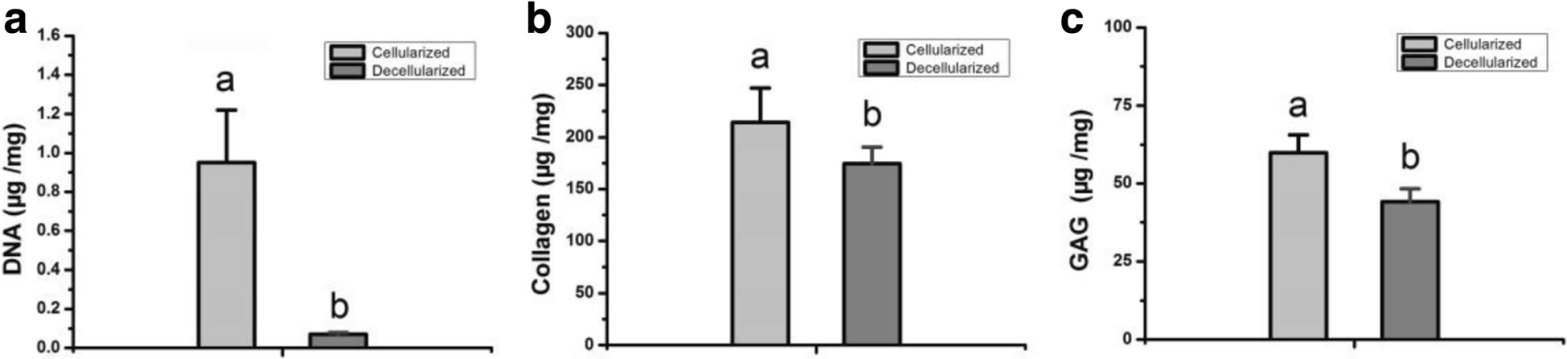
Quantification of DNA, collagen, and GAG in the fibrocartilage bioscaffolds. **a** Content of DNA within the cellularized or decellularized bioscaffolds. **b** Content of collagen within the cellularized or decellularized bioscaffolds. **c** Content of GAG within the cellularized or decellularized bioscaffolds. Dissimilar letters indicating a significant difference (*P < 0.05*)

The quantity of DNA measured for cellularization bioscaffold samples was 0.95 ± 0.27 μg/mg (fibrocartilage dry weight), which was significantly greater (*P* < 0.05) than that for decellularization bioscaffold samples (0.07 ± 0.01 μg/mg) (Fig. 5a), 92.6% DNA is removed during decellularization. The quantity of collagen measured for cellularization bioscaffold samples was 214.16 ± 32.99 μg/mg (dry weight), which was significantly greater (P < 0.05) than that for decellularization bioscaffold samples (174.58 ± 29.67 μg/mg) (Fig. 5b), only 18.5% collagen is lost during decellularization. The quantity of GAG measured for cellularization bioscaffold samples was 59.86 ± 5.7 μg/mg (dry weight), which was significantly greater (*P* < 0.05) than that for decellularization bioscaffold samples (44.11 ± 4.19 μg/mg) (Fig. 5c), only 26.3% GAG is lost during decellularization. These results are consistent with the SR-FTIR examination results.

## Discussion

Decellularized bioscaffold provide a new opportunity for the repair of bone-tendon injury, and quantitative mapping of bioscaffold structure and component information is a crucial prerequisite for further evaluation of bioscaffolds function. In this study, we for the first time applied the SR-FTIR imaging technique to quantitatively map decellularized book-shape fibrocartilage bioscaffolds and compared the observations with those of traditional histological staining techniques and biochemical assay. This technique provided more detailed information including the contents and distribution of collagen fiber and proteoglycan in the decellularized bioscaffolds.

Conventional histological sections, SEM, and 3D X-ray microscopy (X-ray μCT) are commonly used for quantitative mapping of the microscopic morphology of bioscaffolds from 2D to 3D. However, the techniques mentioned above cannot capture changes in the microstructure of the matrix, which limits its further application. Decellularized bioscaffolds, such as the book-shape fibrocartilage bioscaffolds we prepared, may affect extracellular matrix components in addition to affecting tissue morphology following decellularization. Therefore, it is necessary to develop a method that can accurately evaluate matrix components. The conventional FTIR has been used to assess chemical composition of extracellular matrix in osteochondral interface, and have quantitatively mapped the matrix and mineral distribution across this multitissue transition [11–13]. However, the spatial resolution used by these studies is 6.25 μm. Our study has applied SR-FTIR at a higher spatial resolution of 5 μm. High spatial resolution imaging is crucial for biomedical morphology research. The application of SR as a high-brightness source of IR photons has enabled the technique to be applied to analysis at the diffraction limit while preserving a high spectral quality [19]. Using this method, the changes in microstructure can be detected in high resolution, and the composition of the sample tissue can also be determined, which satisfies our requirements.

SR-FTIR has a high SNR and can be used to determine the chemical composition in micron or micro-sample areas, which have been applied for the quantitative mapping of structure and function of stain-free and label-free samples [16, 17]. The mid and far infrared domain would reveal the phonon spectra and electron energy spectra of crystalline and amorphous solids, while the vibration rotation bands exhibit large molecules, including protein, nucleic acid, carbohydrate, lipid, and biological membrane. After we obtain the composition, structure and properties of the material, from the spectra analysis, we can further study the relevant physical phenomena, catalysis process, biochemical reaction and mechanism [19–21]. With the application of the SR-FTIR technique, the distribution of biological components (e.g., collagen and proteoglycan) within the microstructure of decellularized bioscaffolds was observed at the cell size. Such structural chemical information can contribute to the assessment of the extent of decellularization on extracellular matrix, and can be used to evaluate bioscaffolds quality. For the first time, SR-FTIR technology was used to reveal the microstructure of the extracellular matrix of decellularized book-shape bioscaffolds. This technique allows the microscopic structure of book-shape bioscaffolds to be linked to the quality of bioscaffolds.

At the same time, SR-FTIR was employed to analyze the biochemical components and cellular composition of neurodegenerative diseases, and has clearly identified changes in lipids, proteins and nucleic acids in the SH-SY5Y cells of patients with Parkinson’s disease [22]. SR-FTIR also allows observation of the distribution and localization of protein/peptide constituents within a single PLGA microsphere [20], none of which are easy to obtain by conventional analysis methods. The present study analyzed the absorption intensity of collagen and proteoglycan, plotted the chemical maps of the samples, and quantitatively analyzed the content and distribution of extracellular matrix in cellularized or decellularized bioscaffolds. In this study, 19.1% collagen and 27.6% proteoglycan is lost during decellularization. Since the cells are partly composed of proteins and carbohydrates, which have specific absorption bands in the IR frequency domain, we think the loss of collagens and proteoglycans during decellularization process in the present study is not caused by one single factor. In addition to actual collagen loss and proteoglycan loss, cell matrix itself provides contribution to amide I signal and carbohydrate signal could be another possible reason. Following the removal of cells, amide I and carbohydrate intensity also decreased correspondingly.

The SR-FTIR technology is advantageous in sample matrix representation, we would further assess the effect of different decellularization treatment on the decellularized bioscaffolds, and changes of matrix components following decellularization of composited cells in the following studies. A comparison between FTIR and SR-FTIR in assessing the matrix components of biological tissue, which is the next step of this study. The future application of SR-FTIR would be extended to characterizing biological tissues and potentially facilitate the understanding the repair of biological tissue damage.

## Conclusion

In this study, the histological morphology of decellularized bioscaffolds was captured by SR-FTIR imaging, and the content and distribution of collagen and proteoglycan in the extracellular matrix of decellularized book-shape bioscaffolds were quantitatively analyzed. Changes in extracellular matrix components, such as collagen and proteoglycan, were observed following decellularization. However, the microstructure of the scaffold was well preserved. SR-FTIR can be used to quantify the contents and distribution of extracellular matrix components in bioscaffolds.

## Abbreviations

BMSC: Bone marrow stromal cell
OCT: Optimum cutting temperature
SEM: Scanning electron microscopy
SR-FTIR: Synchrotron radiation-based Fourier transform infrared microspectroscopy
SSRF: Shanghai Synchrotron Radiation Facility
